# Predictors of mortality for blunt trauma patients in intensive care: A retrospective cohort study

**DOI:** 10.12688/f1000research.138364.5

**Published:** 2024-12-06

**Authors:** Michael Jennings, James Booker, Amy Addison, Rebecca Egglestone, Ahilanandan Dushianthan

**Affiliations:** 1 General Intensive Care Unit, University Hospital Southampton NHS Foundation Trust, Tremona Road, Southampton, SO16 6YD, UK; 2 Department of Anaesthetics and Perioperative Medicine, University College London Hospitals NHS Foundation Trust, 235 Euston Road, London, NW1 2BU, UK; 3 Department of Neurosurgery, Wessex Neurological Centre, University Hospital Southampton NHS Foundation Trust, Tremona Road, Southampton, SO16 6YD, UK; 4 Faculty of Medicine, University of Southampton, Tremona Road, Southampton, SO16 6YD, UK; 5 NIHR Southampton Clinical Research Facility and NIHR Southampton Biomedical Research Centre, University Hospital Southampton NHS Foundation Trust and University of Southampton, Tremona Road, Southampton, SO16 6YD, UK

**Keywords:** intensive care, critical care, trauma, mortality, scoring systems

## Abstract

**Background:**

Major trauma places substantial demand on critical care services, is a leading cause of death in under 40-year-olds and causes significant morbidity and mortality across all age groups. Various factors influence patient outcome and predefining these could allow prognostication. The aim of this study was to identify predictors of mortality from major trauma in intensive care.

**Methods:**

This was a retrospective study of adult trauma patients admitted to general intensive care between January 2018 and December 2019. We assessed the impact on mortality of patient demographics, patterns of injury, injury scores (Glasgow Coma Score (GCS), Charlson’s comorbidity index (CCI), Acute Physiology and Health Evaluation II (APACHE II), Injury Severity Score (ISS) and Probability of Survival Score (Ps19)), number of surgeries and mechanism of injury using logistic regression.

**Results:**

A total of 414 patients were included with a median age of 54 years (IQR 34–72). Overall mortality was 18.6%. The most common mechanism of injury was traffic collision (46%). Non-survivors were older, had higher ISS scores with lower GCS on admission and lower probability of survival scores. Factors independently predictive of mortality were age 70-80 (OR 3.267, p = 0.029), age >80 (OR 27.043, p < 0.001) and GCS < 15 (OR 8.728, p < 0.001). Ps19 was the best predictor of mortality (p <0.001 for each score category), with an AUROC of 0.90.

**Conclusions:**

The significant mortality predictors were age, fall from <2 metres, injury of head or limbs, GCS <15 and Ps19. Contrary to previous studies, CCI and APACHE II did not significantly predict mortality. Although Ps19 was found to be the best current prognostic score, trauma prognostication would benefit from a single validated scoring system incorporating both physiological variables and injury patterns.

**Abbreviations**
AIS: Abbreviated Injury Severity
APACHE II: Acute Physiology and Chronic Health Evaluation II
AUROC: area under the receiver operator curve
CCI: Carlson Comorbidity Index
CKD: chronic kidney disease
GCS: Glasgow Coma Score
GICU: general intensive care unit
HLOS: Hospital Length of Stay
HRA: Health Research Authority
ICU: intensive care unit
ISS: injury severity score
IRAS: Integrated Research Application System
LOS: length of stay
MTCs: Multi Trauma Centres
NHS: National Health Service
NICU: Neuro Intensive Care unit
Ps19: Trauma Audit and Research Network Probability of Survival Score
RTCs: road traffic collisions
TARN: Trauma Research Audit Network
VAP: ventilator associated pneumonia

## Introduction

Major trauma accounts for almost 10% of all deaths worldwide. ^1^ The National Audit Office estimates that there are more than 20,000 major trauma cases each year in England, resulting in 5,400 deaths. ^2^ Furthermore, the demographics and injury patterns of the major trauma population in the UK are changing. Data from the Trauma Audit and Research Network (TARN) show that there has been an increase in the mean age of trauma patients between 1990 and 2013 (36.1 years to 53.8 years), along with a change in the most common mechanism of injury from road traffic collision (RTC) (59.1%) in 1990, to low fall (39.1%) in 2013. Critically unwell trauma patients typically require admission to the Intensive Care Unit (ICU), frequently making up the most resource-intensive critical care patient group (46.9% of ICU patients in a multicentre US study), ^3^ with significant morbidity and mortality. The burden caused by trauma patients on healthcare systems and the shifts in trauma patient populations has increased the need for evaluating existing scoring systems for their prognostication potential in the ICU trauma population. ^4^

Predicting mortality in the critically unwell trauma patient poses a significant challenge due to the heterogeneity of the patient group and the multitude of patient specific factors that affect ICU outcomes, such as age, comorbidities, and injury patterns. ^5^^–^^10^ Many of these factors have been examined by several previous studies, but there is no consensus on the most useful prognostication scores. Both physiological and anatomical scoring systems have been purported to correlate best with mortality, ^11^^,^^12^ thus there is a current requirement for development of a new scoring tool for early mortality prediction in trauma ICU patients that incorporates a combination of physiological and anatomical scoring components. ^13^^,^^14^

The aim of this investigation was to determine which patient specific factors (present at the point of admission) and which injury severity scoring systems are the most accurate predictors of poor outcome in trauma patients admitted to ICU.

## Methods

### Study design

This is a retrospective study of all critically ill trauma patients aged ≥18 years admitted to the General Intensive Care Unit (GICU) at Southampton General Hospital, between January 2018 and December 2019. ^15^ Major trauma patients were defined as those who had sustained significant injuries due to trauma, resulting in requirement for organ support. Only these patients met the admission requirements for GICU and only GICU patients were included in this study. Patients admitted to other clinical areas including the high dependency unit (HDU) and the neurosciences intensive care unit (NICU) were excluded. These patients did not meet major trauma admission criteria for GICU because they had suffered either minor trauma or isolated neurological trauma, thus they were deemed outside the scope of this study. Penetrating trauma patients were not deliberately excluded, however, our dataset contained only blunt trauma patients as a consequence of the local epidemiology. The sample size was determined by the number patients admitted during the defined time period and was comparable to studies of similar design. Authors did not have access to information that could identify individual participants during or after data collection. Ethical approval was obtained through Ethics and Research Governance Online (ERGO) by the Faculty of Medicine at Southampton University on 4 August 2020, Reference 56519. This study was part of the large CRIT-CO study (Outcomes of Patients Admitted with Critical-Illness to the General Intensive Care Unit – a Retrospective Observational Study) IRAS Reference 232922. This study used retrospective analysis of non-identifiable patient data, thus the need for individual informed consent was waived.

### Baseline data and outcomes

The following variables were collected from all available Southampton General Hospital databases: age, sex, comorbidities, mechanism of injury, and injury distribution. ^15^ The following scores were recorded based on each patient’s condition on admission:

*Glasgow Coma Score (GCS)* – assessment of impairment of conscious level in response to defined stimuli, initially developed for assessment of traumatic brain injury. It has a minimum score of 3 and a maximum score of 15 and is the most widely-used score we evaluated. ^16^

*Injury Severity Score (ISS)* – used to describe severity of injury in trauma patients. Injury severity of each of 6 body systems are scored according the Abbreviated Injury Scale (AIS) 0-6. The three body systems with the highest AIS scores are used to calculate ISS. Each is squared and the sum of these three scores gives the ISS which ranges in value from 3-75. If any system has an 'unsurvivable' injury (AIS = 6), the total score automatically becomes 75. ^17^

*TARN Probability of Survival Score (Ps19)* – an updated composite score based on Trauma Score and Injury Severity Score (TRISS). It incorporates age, sex, trauma type (blunt/penetrating), Revised Trauma Score (RTS), Injury Severity Score (ISS), Glasgow Coma Scale (GCS), number of comorbidities and outcomes at 30 days to give a probability of survival. ^18^

*Acute Physiology and Health Evaluation II (APACHE II) score* – a physiological scoring system that incorporates age, serum laboratory values (pH, sodium, potassium, creatinine, haematocrit, white blood cell count), patient signs (temperature, mean arterial pressure, heart rate, respiratory rate, FiO2), and both acute and chronic diseases (acute renal failure, history of immunocompromise and organ failure). APACHE II is normally used for predicting mortality in ICU patients. The score ranges from 0-71 with increasing score associated with higher mortality. ^19^

*Charlson’s comorbidity index (CCI)* – most widely used comorbidity index. Determines survival rate (1 year and 10 year) in patients with multiple comorbidities. CCI has been adapted multiple times since its inception in 1987 and for the purposes of our study, CCI was determined using an online calculator. ^20^ 0-4 points were assigned for advancing age and between 1 and 6 points are assigned for comorbidities based on their severity including myocardial infarction, congestive heart failure, peripheral vascular disease, cerebrovascular disease, dementia, chronic pulmonary disease, rheumatological disease, peptic ulcer disease, liver disease, diabetes, hemiplegia, paraplegia, renal disease, malignancy, leukaemia, lymphoma and acquired immunodeficiency syndrome (AIDS). Scores were summed to provide a total score to predict mortality. For the multivariate analysis, a modified CCI score was calculated by subtracting the age component of CCI from the total CCI score to avoid confounding between age and CCI.

The outcomes evaluated in our study were duration of mechanical ventilation, ICU and hospital length of stay, and 28-day all cause hospital mortality.

### Statistical analysis

Continuous variables are expressed as median and interquartile range (IQR). Mann Whitney U was used as the statistical analysis for continuous variables and chi-square for categorical variables. The distribution of variables was assessed and if they had a non-normal distribution they were dichotomised into categorical variables with equal sized groups. Univariate analysis using logistic regression to investigate if variables that varied significantly between the survival and non-survival group, were also significant predictors of mortality. Prior to a multivariate analysis a correlation matrix was done to assess the collinearity between each of the significant predictors using Spearman’s test. This informed the subsequent multivariate analysis using a logistic regression to identify independent significant predictors of survival. Predictors were deemed significant if p < 0.05. Additionally, receiver operating characteristics (ROC) area under the curve (AUC) graphs were constructed to assess each variables performance in predicting mortality. Data analysis was done in SPSS Version 25 (RRID:SCR_016479) and RStudio Version 1.4.1103 (RRID:SCR_000432) using packages: dplyr, ggplot2, lme4 and pROC. ^15^

## Results

### Demographics

**Figure 1.**
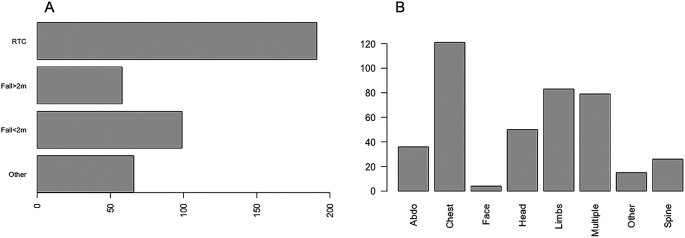
The mechanism of injury and proportion of injuries in different body regions.

A total of 414 critically injured trauma patients were admitted to the Intensive Care Unit between January 2018 and December 2019. Of these, 69.3% (n = 287) were male and 30.7% (n = 127) were female. The median age was 54 years (IQR 34–72). Of those admitted, 66.2% (n = 274) had at least one co-morbidity and the median CCI was 1 (IQR 0–3). The most common mechanism of injury was vehicle incident (46.1%), followed by fall <2 metres (23.9%). The most common body part injured was chest (29.2%), followed by other (20%), multiple injuries (19.1%), head (12.1%), abdominal (8.7%), spinal (6.3%), limbs (3.6%) and facial injuries (1%) ( Figure 1). The median GCS, ISS and APACHE II scores were 15, 22 and 11 respectively.

Overall, the 28-day all cause hospital survival was 81.4% (n = 337), with survivors being on average younger than non-survivors (51 (32–68) vs 74-years-old (55–85). There were no survival differences between male and female patients. Survivors had fewer comorbidities than non survivors (CCI medians of 1 (0–4) and 4, ^1^^–^^4^ respectively). Among non-survivors, fall of <2 metres was the most common mechanism of injury (39%) reflecting the older age of this group, followed by an RTC (32.5%). Among survivors, the most common mechanism of injury was RTC (49.3%), followed by fall from <2 metres (20.5%). Non-survivors had lower GCS at presentation than survivors (9 vs 15). They also had higher ISS (25 vs 20) and higher APACHE-II scores (13 vs 11) than survivors at presentation.

The Ps19 predictive model was significantly lower for non-survivors (59 vs 98). The type of body region injured also varied between survivors and non-survivors. The non-survivors had increased frequency of head injury and the survivors had more chest injuries. Abdominal injuries were more common in survivors than non-survivors (10.1% vs 2.6%), whereas limb injuries were more common in non-survivors than survivors (10.4% and 2.1% respectively) ( Table 1).

**Table 1.** Patient demographics and injury characteristics.

Variable	All patients (n = 414)	Survivors (n = 337)	Non-survivors (n = 77)
Age, years	54 (34–72)	51 (32–68)	74 (55–85)
Gender			
Female	127 (30.7%)	103 (30.6%)	24 (31.2%)
Male	287 (69.3%)	234 (69.4%)	53 (68.8%)
Co-morbidity			
Any	274 (66.2%)	218 (64.7%)	56 (72.72)
CCI median	2 (1-4)	2 (1-4)	4 (2-5)
Mechanism of injury			
Vehicle incident (RTC)	191 (46.1%)	166 (49.3%)	25 (32.5%)
Fall > 2 m	58 (14.0%)	48 (14.2%)	10 (13.0%)
Fall < 2 m	99 (23.9%)	69 (20.5%)	30 (39.0%)
Other	66 (15.9%)	54 (16.0%)	12 (15.6%)
GCS	15 (14–15)	15 (14–15)	9 (3–15)
ISS score	22 (13–29)	20 (12–29)	25 (16–38)
Ps19	97 (86–99)	98 (92–99)	59 (25–85)
APACHE II	11 (7–14)	11 (7–14)	13 (9–18)
Number of surgeries (n)	1 (0–2)	1 (0–2)	0 (0–1)
Most severely injured body region:			
Head	50 (12.1%)	24 (7.1%)	26 (33.8%)
Face	4 (1.0%)	4 (1.2%)	0
Chest	121 (29.2%)	112 (33.3%)	9 (11.7%)
Spine	26 (6.3%)	22 (6.5%)	4 (5.2%)
Abdomen	36 (8.7%)	34 (10.1%)	2 (2.6%)
Multiple	79 (19.1%)	64 (19.0%)	15 (19.5%)
Limbs	15 (3.6%)	7 (2.1%)	8 (10.4%)
Other	83 (20.0%)	70 (20.8%)	13 (16.9%)

Data presented as median and interquartile ranges or number and (%). APACHE II: Acute physiology and chronic health evaluation; CCI: Charlson’s Comorbidity Index; GCS: Glasgow Coma Scale; ISS: Injury Severity Score; Ps19: Probability of survival; RTC: Road Traffic Collision.

We performed univariate logistic regression analysis to assess the association between common variables that demonstrated significant difference between survivor and non-survivor groups with 28-day hospital survival ( Table 2). In the univariate analysis, the following factors were found to be significant predictors of mortality: age (OR 1.04, CI 1.03-1.06, p < 0.001), CCI (OR 1.51, CI 1.32-1.74, p < 0.001, fall <2 metres (OR 3,17, CI 1.74–5.84, p < 0.001), GCS <15 (OR 3.79, CI 2.21–6.63, p < 0.001), ISS 41–60 (OR 3.10, CI 1.46–6.46, p = 0.00269), Ps19 < 81 (p = 0.001), number of surgeries (OR 0.627, CI 0.467–0.806, p < 0.001) and the most severely injured body region of the head (OR 11.1, CI 4.87–27.1, p < 0.001), multiple injuries (OR 2.60, CI 1.12–6.31, p = 0.0288) and other injuries (OR 12.7, 95% CI (3.84–44.0, p = 0.001) ( Table 2). A multivariate analysis was conducted using these variables which demonstrated that age 70-80 (OR 3.267, CI 1.102-9.404, p = 0.029), age >80 (OR 27.043, CI 10.228-78.264, p < 0.001) and GCS < 15 (OR 8.728, CI 4.273-19.504, p < 0.001), were independent predictors of mortality ( Table 3). Modified CCI, fall <2 metres, and number of surgeries were not found to independently predict mortality in the multivariate analysis.

**Table 2.** Univariate analysis of variables that significantly differed between survivor and non-survivor groups using logistic regression to predict mortality.

Predictor	Estimate	Standard error	Z value	P value	OR (CI)
Age	0.0437	0.00710	6.15	<0.001 ***	1.04 (1.03-1.06)
Charlson Comorbidity Index	0.414	0.0.071	5.849	<0.001 ***	1.51 (1.32-1.74)
Mechanism of injury • RTC • Fall>2 m • Fall<2 m • Other	0 ^a^ 0.371 1.15 0.436	0 ^a^ 0.411 0.308 0.387	0 ^a^ 0.905 3.75 1.13	0 ^a^ 0.366 <0.001 *** 0.260	0 ^a^ 1.45 (0.624-3.17) 3.17 (1.74-5.84) 1.55 (0.705-3.25)
Glasgow Coma Score (GCS) • 15 • <15	0 ^a^ 1.33	0 ^a^ 0.279	0 ^a^ 4.77	0 ^a^ <0.001 ***	0 ^a^ 3.79 (2.21-6.63)
Injury Severity Score (ISS) • 1-20 • 21-40 • 41-60 • 61-80	0 ^a^ 0.541 1.13 1.89	0 ^a^ 0.285 0.378 1.02	0 ^a^ 1.90 3.00 1.85	0 ^a^ 0.0580 0.00269 ** 0.0645	0 ^a^ 1.72 (0.985-3.02) 3.10 (1.46-6.46) 6.62 (0.767-57.1)
Probability of Survival (Ps19) • 81-100 • 61-80 • 41-60 • 21-40 • 0-20	0 ^a^ 2.18 3.51 2.81 5.33	0 ^a^ 0.420 0.569 0.549 1.05	0 ^a^ 5.18 6.17 5.12 5.06	0 ^a^ <0.001 *** <0.001 *** <0.001 *** <0.001 ***	0 ^a^ 8.81 (3.82-20.1) 33.5 (11.6-112) 16.5 (5.68-50.3) 206 (39.4-3800)
APACHE II • 0-10 • 11-20 • 21-30	0 ^a^ 0.648 0.638	0 ^a^ 0.406 0.701	0 ^a^ 1.60 0.910	0 ^a^ 0.110 0.363	0 ^a^ 1.91 (0.874-4.36) 1.89 (0.399-6.83)
Number of surgeries	-0.466	0.139	-3.35	0.001 ***	0.627(0.467-0.806)
Most severely injured body region • Chest • Head • Face • Spine • Abdomen • Multiple • Limbs • Other	0 ^a^ 2.41 -13.2 0.702 -0.426 0.956 0.723 2.54	0 ^a^ 0.435 728 0.636 0.799 0.437 0.448 0.614	0 ^a^ 5.54 -0.018 1.10 0.533 2.19 1.62 4.14	0 ^a^ <0.001 *** 0.986 0.270 0.594 0.0288 * 0.106 0.001 ***	0 ^a^ 11.1 (4.87-27.1) 0 (0-2x10 ^30^) 2.02 (0.517-6.66) 0.653 (0.0973-2.63) 2.60 (1.12-6.31) 2.06 (0.861-5.07) 12.7 (3.84-44.0)

* p < 0.05.

** p < 0.01.

*** p < 0.001.

0 ^a^, Reference category.

**Table 3.** Multivariate analysis of variables that were significant in the univariate analysis that were not prediction variables. Analysis done using logistic regression predicting mortality.

Predictor	Estimate	Standard error	Z value	P value	OR (CI)
Age • <50 • 50-60 • 60-70 • 70-80 • >80	0 ^a^ 1.010 0.663 1.184 3.297	0 ^a^ 0.523 0.576 0.541 0.517	0 ^a^ 1.929 1.153 2.187 6.382	0 ^a^ 0.090 0.284 0.029 * <0.001 ***	0 ^a^ 2.744 (0.955-7.590) 1.941 (0.594-5.484) 3.267 (1.102-9.404) 27.043 (10.228-78.264)
Modified Charlson’s Comorbidity Index (CCI)	-0.002	0.226	-0.007	0.994	0.998 (0.627-1.538)
Glasgow Coma Score (GCS) • 15 • <15	0 ^a^ 2.167	0 ^a^ 0.384	0 ^a^ 5.643	0 ^a^ <0.001 ***	0 ^a^ 8.728 (4.273-19.504)
Mechanism of injury • RTC • Fall>2 m • Fall<2 m • Other	0 ^a^ 0.164 0.512 0.840	0 ^a^ 0.489 0.419 0.481	0 ^a^ 0.337 1.224 1.746	0 ^a^ 0.736 0.221 0.081	0 ^a^ 1.179 (0.435-3.007) 1.670 (0.733-3.814) 2.316 (0.886-5.922)
Number of surgeries	-0.123	0.131	-0.940	0.347	0.884 (0.667-1.122)

* p < 0.05.

** p < 0.01.

*** p < 0.001.

0 ^a^, Reference category.

**Figure 2.**
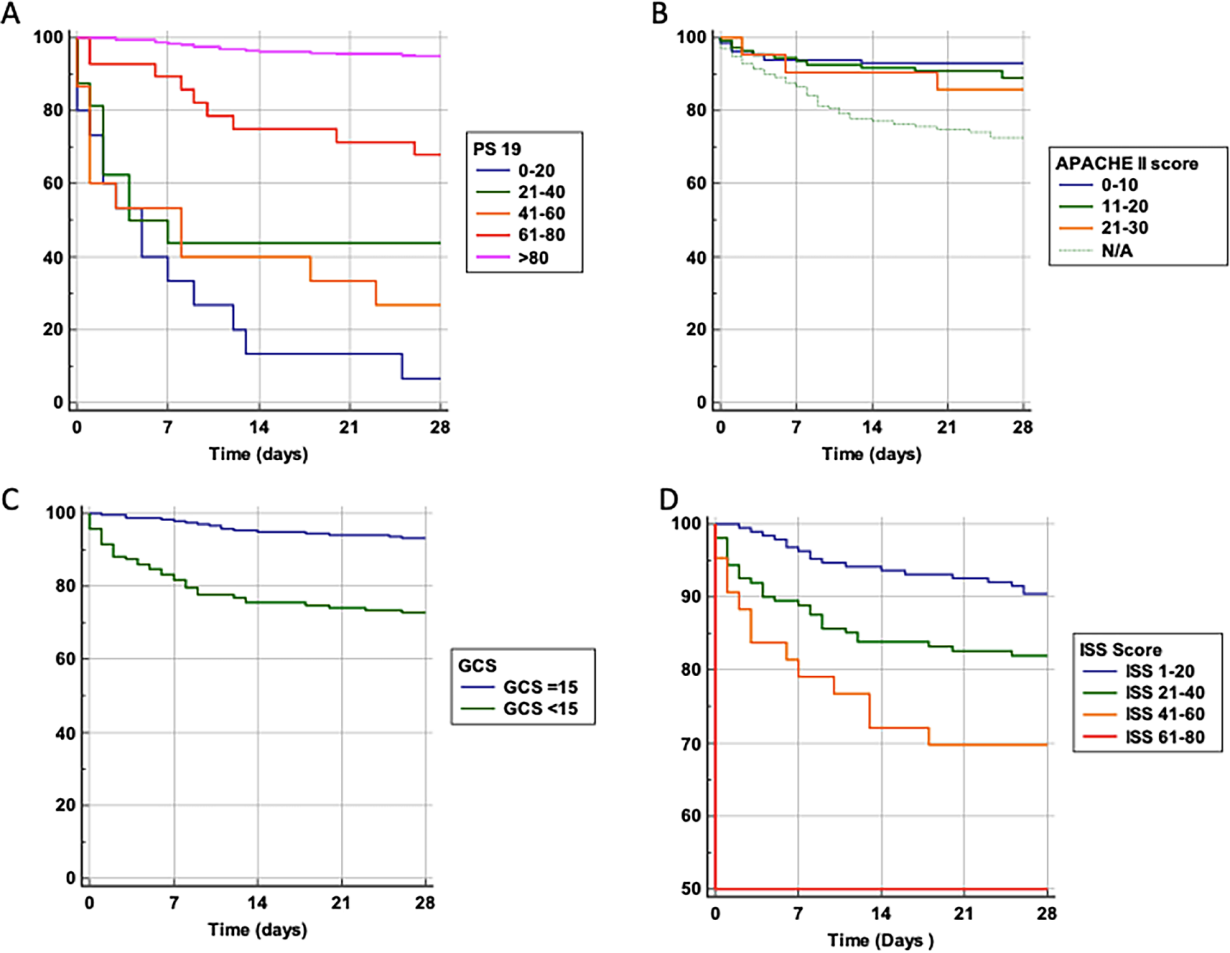
Survival probability and trauma scoring systems. Probability of survival against total number of days in hospital. Patients are categorised into groups based on their scores in scoring systems. A: Ps19 Survival probability. B: APACHE II Survival probability. APACHE II scores could not be calculated for 139 patients, so this is taken into consideration with a N/A line. One patient was excluded from the APACHE II graph due to being the only one who had a score >30. C: GCS Survival probability. D: ISS Survival probability. APACHE II: Acute physiology and chronic health evaluation; GCS: Glasgow coma scale; ISS: Injury severity score; Ps19: Probability of survival.

We performed a probability of survival analysis based on variables including age and the scoring systems Ps19, ISS, GCS, and APACHE-II ( Figure 2). For Ps19 ( Figure 2A), patients with a low Ps19 0–20 had an almost linear decrease in survival probability up until 14 days, had a lower survival probability and were more likely to die sooner. Patients with Ps19 scores between 21–60 had similar survival probabilities until day seven, at which point they diverge with the 41–60 group having the lowest survival probability at 28 days (28%), and the 21–40 group having a survival probability of 44%. Patients with Ps19 scores of 61 or higher had significantly higher probability of survival than the other groups, with the 61–80 group demonstrating more than 70% chance of survival at 28 days. The Ps19 score >80 group demonstrated a survival probability of over 90%.

For APACHE II score, likelihood of survival at 28 days decreased with increasing APACHE II scores ( Figure 2B). Until the seventh day there was a similar survival curve for all APACHE II scores groups, after which patients with a score of 0–10 clearly show a higher probability of survival compared to patients with an APACHE II score of 11–20 or 21–30, (92%, 89% and 87% at 28 days, respectively).

Patients with reduced GCS ( Figure 2C), had lower likelihood of survival compared to those admitted GCS 15 (73% and 94% at 28 days respectively).

For ISS scores ( Figure 2D), patients in the highest score range (61–80) had only a 50% chance of survival at 28 days. Patients with a score of 41–60 had a 70% survival probability, those scoring 21–40 had an 84% chance of survival whereas the group scoring 1–20 had a 90% probability of survival. Thus, a higher ISS score was associated with a lower probability of survival.

**Figure 3.**
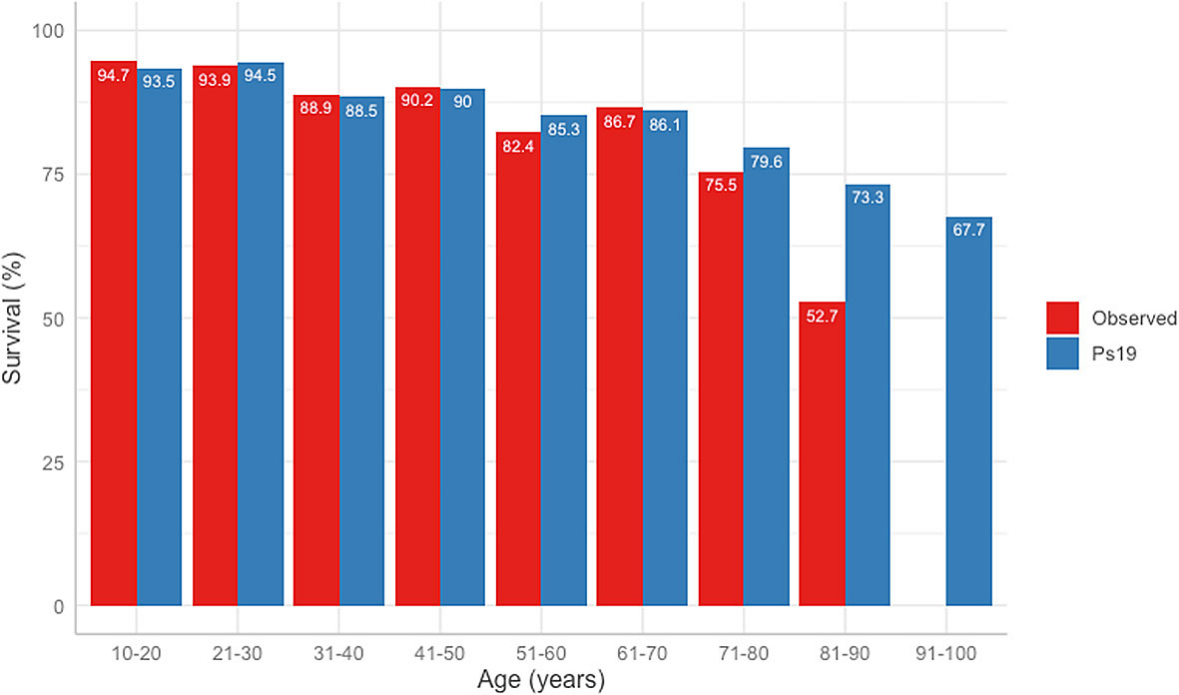
Observed vs Ps19 predicted survival in different age groups.

The difference between the predicted Ps19 and observed mortality for the cohort is shown in Figure 3. The Ps19 predicted score was similar to the expected mortality for most ages, except for the groups >80 years of age ( Figure 3).

**Figure 4.**
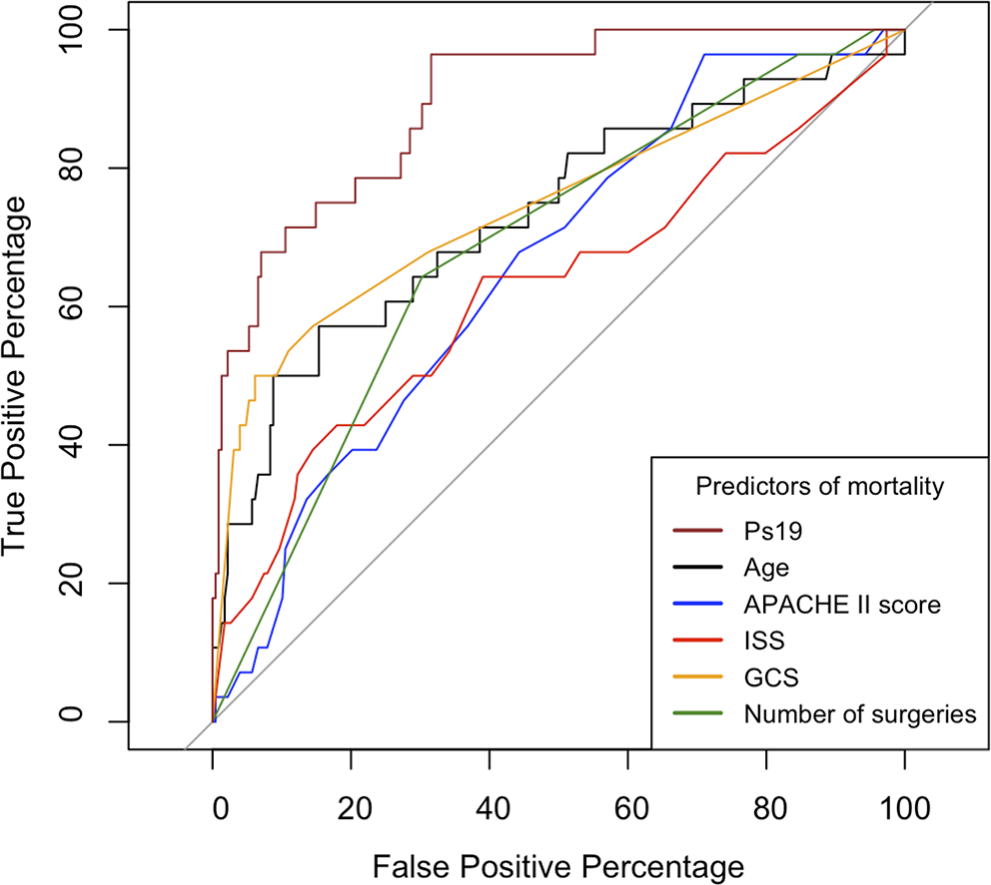
ROC curves comparing predictors of mortality. Calculated for APACHE II Score, ISS, GCS, Ps19 and Number of surgeries in mortality correlation prediction. ROC: receiver operator characteristic, AUROC: area under the receiver operator curve, APACHE II: Acute physiological assessment and chronic health evaluation, ISS: Injury Severity Score, GCS: Glasgow Coma Scale, Ps19: Probability of Survival Score.

The area under the receiver operator curve (AUROC) was statistically significant for all variables ( Figure 4). Ps19 was the best predictor of mortality with an AUROC of 0.90 (95% CI 0.85–0.96) followed by GCS AUROC of 0.75 (95% CI 0.64–0.86) and age 0.73 (95% CI 0.62–0.85). ISS, APACHE II and number of surgeries were less predictive of mortality in comparison to these variables with ISS being the worst predictor with an AUROC of 0.66 (95% CI 0.50–0.76).

Overall, 190 patients (45.9%) required invasive mechanical ventilation and the proportion was higher in non-survivors, compared with survivors (68.8% vs 40.7%) and for both groups the duration of mechanical ventilation was three days. Median ICU and hospital length stay were 3 (IQR 1, 7) and 13 (IQR 7, 26) respectively. There was no statistically significant difference between the ICU length of stay (LOS) for survivors and non-survivors; however, survivors had a longer hospital LOS (15 vs 7 days p < 0.01) ( Table 4).

**Table 4.** Duration of mechanical ventilation, ICU and hospital length of stay. Data presented as median and interquartile ranges.

Outcome	All patients	Survivors	Non-survivors	p-value
Mechanical ventilation days	3 (2, 6)	3 (2, 6)	3 (2, 6)	0.772
ICU length of stay (days)	3 (1, 7)	3 (1, 7)	3 (1, 6)	0.430
Hospital length of stay (days)	13 (7, 26)	15 (9, 27)	7 (2,18)	<0.001 ***

* p < 0.05.

** p < 0.01.

*** p < 0.001.

## Discussion

This study evaluated different patient specific and injury specific factors that influence hospital mortality in critically ill blunt trauma patients. Patient specific factors we investigated included age, gender, and pre-existing comorbidities. Increased age was unsurprisingly found to be associated with a higher mortality. This association is likely to be multifactorial due to an increased risk of frailty, higher likelihood of under triage, and altered physiological mechanisms in elderly patients. Moreover, older patients may have fallen due to an intracranial neurological reason, and are also more likely to develop complications from a long-lie. Older patients have increased comorbidities with concurrent risk of polypharmacy including anticoagulant medication, which further increases risk of adverse outcome from trauma.

Presence of comorbidities in our cohort as determined by CCI was found to be predictive of mortality in the univariate analysis, however modified CCI was not found to be predictive of mortality in the multivariate analysis. This suggests the predictive power of CCI was dominated by the age component. In contrast to our findings, previous studies have found a direct impact of CCI on mortality. ^13^ Whilst some studies have given conflicting evidence as to the effect of comorbidities on hospital length of stay, ^21^^,^^22^ their importance is acknowledged by their inclusion in trauma scoring systems such as the Ps19 model. In our study, only age and GCS were found were associated with increased hospital mortality in critically ill trauma patients in the multivariate model.

Injury specific factors such as mechanism of injury, body region injured, and severity of injury were all found to affect mortality in our univariate analyses. In our cohort, most deaths were due to head injuries or polytrauma. Falls and RTC were the most common mechanism of injury, which is consistent with published data from both the UK and the USA. ^23^^,^^24^ The overall mortality in our cohort was 18.6%, with an increased mortality in patients with a fall from <2 metres. Of those injured in an RTC, 86.9% survived compared to 69.7% of those who fell <2 metres. 2 metres was used as a cut-off because falls of >2 metres are considered by NHS England a sufficient mechanism to activate major trauma responses and divert patients to a Major Trauma Centre. ^25^ It appeared counterintuitive that patient falls <2 metres had worse outcomes. However, this finding was not significant in the multivariate model and can be explained by a higher age in this cohort. Older patients are more likely to have severe injuries from <2 metres and subsequent ICU admission. This is consistent with national data from the TARN database. ^26^ In our multivariate models, no injury specific factors were found to be an independent predictor for mortality, which is consistent with findings from previous studies. ^12^^,^^27^

Two recent single-centre retrospective observational studies found the following factors to be associated with increased mortality in ICU from trauma: age >60 years, comorbidities (CCI), severity of trauma (New Injury Severity Score (NISS) and Revised Injury Severity Classification (RISC)), patient severity (APACHE II), traumatic brain injury, the use of mechanical ventilation, renal dysfunction in the first 24 hours, and the use of vasoactive drugs and circulatory complications. ^28^^,^^29^ In contrast to our findings, the scores most highly predictive for mortality were APACHE II and NISS. An Australian meta-analysis of over 5000 patients across 25 centres demonstrated similar findings but also showed the Australian and New Zealand Risk of Death (ANZROD) mortality prediction model and APACHE III to be superior to the anatomical scoring systems for mortality prediction (e.g., ISS and NISS). ^13^

Although a higher APACHE II score and CCI were more commonly found in the non-survivor group in our study, these associations proved inadequate predictors of mortality in univariate analyses. However, our findings agreed with previous work that an increased ISS and decreased Ps19 both predict increased mortality. ^27^^,^^29^^–^^31^ Ps19 was our best performing mortality predictor with an AUROC of 0.9 (95% Cl 0.85–0.96), outperforming ISS (the most used scoring tool).

Our study presents significant limitations. Firstly, the dataset was collected retrospectively from a single centre, notably excluding patients with primary head injury. Secondly, our study did not extensively examine indicators of patient morbidity following trauma; for example, renal dysfunction and ICU interventions such as other organ support measures including renal replacement therapy and the use of vasopressors. We also did not assess other important outcomes such as lasting neurological deficits, rehabilitation required following discharge, which may have provided further context for mortality prediction analyses. We did not include some trauma scores which other authors have found valuable, such as the calculated Revised Trauma Score (cRTS), analysis of which would have produced a more exhaustive study. Finally, the outcome of our study was limited to 28-day mortality and does not report mortality data at longer timepoints. Whilst there are logistical challenges with data collection over extended timeframes in ICU patients following their discharge, the decision to limit the mortality window to 28 days limits the scope of conclusions to prognostication within a short pre-defined window. Nevertheless, our study complements the existing literature with noteworthy analysis of the mortality prediction capability of a range of scoring systems including ICU specific scoring systems and presents comparable sample sizes to similar recent single-centre studies. ^28^^,^^29^ It was also noted that neither of these studies included the Ps19 scoring system, which is currently used by the TARN (UK), which we found to be the most highly predictive of mortality from trauma in ICU.

## Conclusions

This study shows that various internal and external factors determine the mortality of ICU patients within a single-centre general ICU. The most significant independent predictors of mortality were age, and GCS. Ps19 and ISS were also found to be useful scores for mortality prediction in our probability of survival analyses. Ps19 was the best performing score overall for mortality prediction. Contrary to previous studies, we did not demonstrate a strong association between mortality and the CCI and APACHE II scoring systems. Our study findings suggest helpful scoring systems, however, currently no scoring system is exhaustive and larger studies exploring multiple component of age, patient characteristics, injury types and frailty may be useful for trauma prognostication. An all-inclusive single validated scoring system incorporating physiological variables, injury patterns and ICU variables could mitigate extended ICU stays by ensuring that interventions patients receive are better tailored to their individual physiological profile and possibly reduce overall mortality in ICU trauma patients. Future studies would benefit from inclusion of morbidity indicators to provide context for the quality of life experienced by survivors of major trauma.

## Data Availability

Zenodo: Underlying data for ‘Predictors of mortality for major trauma patients in intensive care: A retrospective cohort study’, https://doi.org/10.5281/zenodo.14218022. ^15^ This project contains the following underlying data:
• Data sheet 2018–2019 29.09.20.xlsx • Trauma analysis.pptx • Ps19 vs survival.tiff • ROCs.tiff • traumadata.csv STROBE checklist for ‘Predictors of mortality for major trauma patients in intensive care: A retrospective cohort study’, https://doi.org/10.5281/zenodo.14218022. ^15^ Data are available under the terms of the Creative Commons Zero “No rights reserved” data waiver (CC0 1.0 Public domain dedication).

## Software availability

Source code available from: https://github.com/m-a-jennings/Predictors-of-Mortality-for-Major-Trauma-Patients-in-Intensive-Care-A-Retrospective-Cohort-Study/

Archived source code at time of publication: https://doi.org/10.5281/zenodo.14218022. ^15^

Data are available under the terms of the Creative Commons Zero “No rights reserved” data waiver (CC0 1.0 Public domain dedication).
