# Supplementary material for: Predictors of mortality for blunt trauma patients in intensive care: A retrospective cohort study
Source: F1000Res. 2024 Dec 6;12:974. Originally published 2023 Aug 14. [Version 5] doi: 10.12688/f1000research.138364.5 (PMC11628930; doi:10.12688/f1000research.138364.5)
Supplement: Peer review history

# Reviewer report: Reviewer response for version 5

**DOI:** 10.5256/f1000research.174997.r346596

**Published:** 2024-12-09

**Authors:** Neta Cohen

**Affiliations:** 1 Tel Aviv Sourasky Medical Center, Tel Aviv University, Tel Aviv, Israel

The authors much improved their methods and I have no more comments.

Is the work clearly and accurately presented and does it cite the current literature?

Yes

If applicable, is the statistical analysis and its interpretation appropriate?

Yes

Are all the source data underlying the results available to ensure full reproducibility?

Yes

Is the study design appropriate and is the work technically sound?

Yes

Are the conclusions drawn adequately supported by the results?

Partly

Are sufficient details of methods and analysis provided to allow replication by others?

Partly

Reviewer Expertise:

NA

I confirm that I have read this submission and believe that I have an appropriate level of expertise to confirm that it is of an acceptable scientific standard.

# Reviewer report: Reviewer response for version 4

**DOI:** 10.5256/f1000research.172463.r328484

**Published:** 2024-10-11

**Authors:** Neta Cohen

**Affiliations:** 1 Tel Aviv Sourasky Medical Center, Tel Aviv University, Tel Aviv, Israel

CCI reflects both patients age and comorbidities, hence, CCI and age are cofactors. The authors removed CCI from tha analysis, but it was not what I meant.

 CCI is an important factor as it reflects both age and chronic disease. Hence , the authors need   to remove points from CCI of each one of the patients, which were given due to advanced age (0-4 points are assigned for advancing age). In this way, the authors will have the ability to explore age and chronic disease separately: they will  create 2 separate different parameters:  1. age  2. Comorbidities. Next,  they can insert both age and comorbidity to the multivariable model to see if they may be independent predictors for mortality.

 The other option (more easy, less accurate)  is to insert only CCI (and not age) to the multivariable analysis, but then we could not know if the comorbidity or age are responsible for the outcome - it can be discussed in the discussion.

 In any way: to remove CCI from the analysis is not acceptable as it is a very important parameter for mortality...

Is the work clearly and accurately presented and does it cite the current literature?

Yes

If applicable, is the statistical analysis and its interpretation appropriate?

Yes

Are all the source data underlying the results available to ensure full reproducibility?

Yes

Is the study design appropriate and is the work technically sound?

Yes

Are the conclusions drawn adequately supported by the results?

Partly

Are sufficient details of methods and analysis provided to allow replication by others?

Partly

Reviewer Expertise:

NA

I confirm that I have read this submission and believe that I have an appropriate level of expertise to confirm that it is of an acceptable scientific standard, however I have significant reservations, as outlined above.

# Response

**Published:** 2024-11-25

**Authors:** Michael Jennings

**Affiliations:** Anaesthetics, UCLH, University College London, London, England, UK

Dear Dr Cohen,

 Thank you for your comments. I hope you will find our changes suitable. We have recalculated CCI values for the univariate analysis and calculated a modified CCI variable removing the age component for the multivariate analysis as you have suggested. This has resulted in new values in tables 1, 2 and 3. We have therefore modified the following sections of the manuscript:

 Abstract - updated to include the newly calculated odds ratios.

 Methods - updated to include explanation of a modified CCI score which removes the age component of CCI for the multivariate analysis.

 Results - Table 1 - CCI medians were recalculated. CCI values were recalculated and updated in the univariate analysis and in Table 2. Modified CCI was added to the multivariate analysis section and added to Table 3.

 Discussion - Explanation added for significance of CCI in univariate analysis but not multivariate analysis.

 New dataset added to online repository and references updated.

 Best wishes,

# Reviewer report: Reviewer response for version 3

**DOI:** 10.5256/f1000research.169909.r313280

**Published:** 2024-08-27

**Authors:** Neta Cohen

**Affiliations:** 1 Tel Aviv Sourasky Medical Center, Tel Aviv University, Tel Aviv, Israel

I hope this time authors will address the critical issues.:

 1. Cci definition

 2. Cci and age are co factors and can not be analysed together in this form

 3. Major trauma definition to understand the inclusion criteria

 Minor comments are :

 1. Fall from 2 meters definition in the methods, 2. Definition of all severity scores 

  3. Insert ps 19 to the roc curve figure to see its performance compared to other.

Is the work clearly and accurately presented and does it cite the current literature?

Yes

If applicable, is the statistical analysis and its interpretation appropriate?

Yes

Are all the source data underlying the results available to ensure full reproducibility?

Yes

Is the study design appropriate and is the work technically sound?

Yes

Are the conclusions drawn adequately supported by the results?

Partly

Are sufficient details of methods and analysis provided to allow replication by others?

Partly

Reviewer Expertise:

NA

I confirm that I have read this submission and believe that I have an appropriate level of expertise to confirm that it is of an acceptable scientific standard, however I have significant reservations, as outlined above.

# Response

**Published:** 2024-09-24

**Authors:** Michael Jennings

**Affiliations:** Anaesthetics, UCLH, University College London, London, England, UK

Dear Dr Cohen, 

 I hope you will approve of the changes we have made to the manuscript for V4 and will now be able to approve its publication and indexing. We have taken care to address your concerns in turn. The changes we have made are as follows:

 1) GCS, ISS, Ps19, APACHE II and CCI have now been defined in the methods section and appropriate references added. 

 2) Major trauma for the purposes of this study has been defined in the paragraph on study design in the methods section.

 3) CCI has been removed from the multivariable analysis (Table 3) because it was a confounder. The results section and the abstract have been updated to reflect this new data in table 3.

 4) Ps19 was added to the ROC curves in figure 4 to compare its performance against other scoring systems.

 5) Figure legends for figure 3 and 4 were switched - this was a mistake in the previous version and the figure legend for figure 4 has been altered to better reflect the contents of the figure.

 I hope you are willing to approve the updated manuscript.

 Best wishes

# Reviewer report: Reviewer response for version 2

**DOI:** 10.5256/f1000research.167939.r295150

**Published:** 2024-07-03

**Authors:** Adnan Özpek

**Affiliations:** 1 Umraniye Training and Research Hospital, University of Health Sciences, İstanbul, Turkey

Dear Editor,

 I do not have any additional revision suggestions regarding the article. It can be indexed in its current form.

 Best Regards,

 Dr.Adnan Özpek

Is the work clearly and accurately presented and does it cite the current literature?

Partly

If applicable, is the statistical analysis and its interpretation appropriate?

I cannot comment. A qualified statistician is required.

Are all the source data underlying the results available to ensure full reproducibility?

Partly

Is the study design appropriate and is the work technically sound?

Partly

Are the conclusions drawn adequately supported by the results?

Partly

Are sufficient details of methods and analysis provided to allow replication by others?

Partly

Reviewer Expertise:

General Surgery, Trauma and Emergency Surgery

I confirm that I have read this submission and believe that I have an appropriate level of expertise to confirm that it is of an acceptable scientific standard.

# Reviewer report: Reviewer response for version 2

**DOI:** 10.5256/f1000research.167939.r295151

**Published:** 2024-07-02

**Authors:** Neta Cohen

**Affiliations:** 1 Tel Aviv Sourasky Medical Center, Tel Aviv University, Tel Aviv, Israel

My main additional comments to the authors are:

 1. A. Please test in your univariable analysis the parameter " CCI" (either as any or as continious variable).

 B. Please explain what is CCI and how did you calculate it, and if you took age >60 etc as one of the parameters to calulate the CCI.

 C. Due to its imporatnace, even if it is not significant in the univariable analysis, you can still test it in your multivariable model. To improve the sensitivity of mulktivariable models I reccomend either to insert any clinical important variable (according to the authors decision and based on the literature) OR to rely on the univariable findings but with higher P value (0.1 is acceptable). Personally I prefer the first method.

 2. With respect to fall< 2 meters - if it is not significant in the multivariable model - **so it is not significant and doesnt merit any further details.** you can simply conclude that **no mechanism was independent predictor for severity in your analysis **, and to cite relevant literature with similar findings. Mechanism is known from previous studies to be poorly associated with injury severity.

Is the work clearly and accurately presented and does it cite the current literature?

Yes

If applicable, is the statistical analysis and its interpretation appropriate?

Yes

Are all the source data underlying the results available to ensure full reproducibility?

Yes

Is the study design appropriate and is the work technically sound?

Yes

Are the conclusions drawn adequately supported by the results?

Partly

Are sufficient details of methods and analysis provided to allow replication by others?

Partly

Reviewer Expertise:

NA

I confirm that I have read this submission and believe that I have an appropriate level of expertise to confirm that it is of an acceptable scientific standard, however I have significant reservations, as outlined above.

# Response

**Published:** 2024-07-31

**Authors:** Michael Jennings

**Affiliations:** Anaesthetics, UCLH, University College London, London, England, UK

Dear Dr Cohen,

 Thank you for your comments on the latest version of the manuscript. In line with your suggestions, for the new version CCI is now included within the univariate and multivariate analyses. (Tables 2 and 3)

 Your other comments have been addressed within the body of the new manuscript.

 Many thanks,

 Dr Mike Jennings

# Response

**Published:** 2024-08-01

**Authors:** Michael Jennings

**Affiliations:** Anaesthetics, UCLH, University College London, London, England, UK

Updates from version 2 (v2) to version 3 (v3) are detailed as follows:

 Abstract: 

 Results section has been updated to include new values from the multivariate regression.

 Conclusion section has been updated to remove fall < 2m and injury to the head and limbs as significant mortality predictors because these were not significant in the new multivariate analysis.

 Methods:

 A description of how CCI was calculated has been added 

 Results:

 Multivariate analysis section has been updated to include CCI as one of the variables

 Table 2 has been reformatted to fix an issue with alignment.

 Table 3 has been changed to include CCI as one of the variables tested

 Discussion:

 The section on age as a significant mortality indicator has been reworded to include parts of the discussion that originally referred to falls < 2m. This was in line with Dr Cohen’s suggestions that we should not place emphasis on falls <2 predicting mortality, given that this finding was not significant in the multivariate analysis. Further explanation of this has been added when we refer to age as a confounding factor in the falls < 2m paragraph.

 Conclusion:

 Fall from a height of <2m and injury of head and limbs have been removed from the conclusion in line with our latest multivariate analysis

# Reviewer report: Reviewer response for version 1

**DOI:** 10.5256/f1000research.151554.r266548

**Published:** 2024-05-21

**Authors:** Neta Cohen

**Affiliations:** 1 Tel Aviv Sourasky Medical Center, Tel Aviv University, Tel Aviv, Israel

This is a retrospective study of all critically ill trauma patients aged ≥18 years admitted to the General Intensive Care Unit (GICU), aimed to determine which patient specific factors (present at the point of admission) and which injury severity scoring systems are the most accurate predictors of poor outcome in trauma patients admitted to ICU.

 Such similar studies were performed earlier (Cohen, N., et.al., 2023 (Ref 1) However, considering the importance of the topic, and the appropriate design, I believe it merit publication.

 Comments:

 1. Why did you exclude patients from high dependency units and neuroscience intensive care unit?

 2. CCI and AGE are co-factors, as age >60 is one of the factors including in the CCI. Try to examine each one separately in the logistic regression model. Please add it in addition to your limitation.

 3, How do you explain falling from height< 2 meters to be more severe than fall > 2 meters? how did you decide about cut-off of 2 meters ( the usual cutoff is 3 meters).?

 4. Please explain better what are the implication of your findings?

Is the work clearly and accurately presented and does it cite the current literature?

Yes

If applicable, is the statistical analysis and its interpretation appropriate?

Yes

Are all the source data underlying the results available to ensure full reproducibility?

Yes

Is the study design appropriate and is the work technically sound?

Yes

Are the conclusions drawn adequately supported by the results?

Partly

Are sufficient details of methods and analysis provided to allow replication by others?

Partly

Reviewer Expertise:

pediatric emergency medicine, emergency medicine, trauma

I confirm that I have read this submission and believe that I have an appropriate level of expertise to confirm that it is of an acceptable scientific standard, however I have significant reservations, as outlined above.

## References

[1] : Refining triage practices by predicting the need for emergent care following major trauma: the experience of a level 1 adult trauma center. *Eur J Trauma Emerg Surg* .2023;49(4) : 10.1007/s00068-022-02195-4 1717-1725 10.1007/s00068-022-02195-4 36522466

# Response

**Published:** 2024-06-13

**Authors:** Michael Jennings

**Affiliations:** Anaesthetics, UCLH, University College London, London, England, UK

Thank you very much for your comments. The manuscript has been amended to address them and we have now submitted an updated version.

 1) Exclusion of patients from the high dependency unit and the neuroscience intensive care unit has been further explained within the methods section. 

 2) To address your comments that CCI and age are cofactors, the univariate and multivariate analyses in tables 2 and 3 have been reanalysed with age as a categorical variable. 

 3) Falls of less than 2 meters were found to be a significant predictor of mortality in the univariate analysis, but not the multivariate analysis. The explanation of this finding has been added to the discussion section along with an explanation for why the cut-off of two meters was chosen.

 4) This discussion and conclusion sections of the manuscript have been improved to explain the implications of our findings in more detail.

 Many thanks

# Reviewer report: Reviewer response for version 1

**DOI:** 10.5256/f1000research.151554.r246878

**Published:** 2024-03-01

**Authors:** Adnan Özpek

**Affiliations:** 1 Umraniye Training and Research Hospital, University of Health Sciences, İstanbul, Turkey

I congratulate the authors for their study. My suggestions for the study are as follows:

 1- It appears that all the patients in the study were blunt trauma patients, which should be noted in the title of this article. If patients with penetrating trauma are included in the study, it should be stated.

 2- Calculated Revised Trauma Score (cRTS) also can be used in addition to the trauma scoring systems used in the study.

 3- In the study, mortality rates for falls below 2 meters were significantly higher than for falls above 2 meters. This is not an expected result. It should be questioned whether the patients who fell below 2 meters fell due to an intracranial neurological reason.

Is the work clearly and accurately presented and does it cite the current literature?

Partly

If applicable, is the statistical analysis and its interpretation appropriate?

I cannot comment. A qualified statistician is required.

Are all the source data underlying the results available to ensure full reproducibility?

Partly

Is the study design appropriate and is the work technically sound?

Partly

Are the conclusions drawn adequately supported by the results?

Partly

Are sufficient details of methods and analysis provided to allow replication by others?

Partly

Reviewer Expertise:

General Surgery, Trauma and Emergency Surgery

I confirm that I have read this submission and believe that I have an appropriate level of expertise to confirm that it is of an acceptable scientific standard, however I have significant reservations, as outlined above.

# Response

**Published:** 2024-06-13

**Authors:** Michael Jennings

**Affiliations:** Anaesthetics, UCLH, University College London, London, England, UK

Thank you very much for your comments. They have been addressed in the new updated manuscript.

 1) The article has been amended to include stipulation that only blunt trauma patients were included. See changes to title, abstract and methods section.

 2) Mention of cRTS has been added to the limitations section of the discussion as an example of useful scoring systems that were omitted. 

 3) Factors contributing to our finding that falls < 2 metres being associated with higher mortality have been discussed in more detail in the discussion section.
